# A Hybrid Deep Learning Framework for Multi-Sensor PMSM Fault Diagnosis Based on SVMD Denoising and Spatiotemporal Feature Fusion

**DOI:** 10.3390/s26175537

**Published:** 2026-08-31

**Authors:** Mingdong Guan, Yiming Peng, Yingxi Xie

**Affiliations:** 1 School of Mechanical and Automotive Engineering, South China University of Technology, Guangzhou 510641, China; guanmingdong1117@163.com; 2 Guangdong GausSense Technology Co., Ltd., Foshan 528200, China; 3 Guangzhou Power Supply Bureau, Guangdong Power Grid Co., Ltd., China Southern Power Grid, Guangzhou 510000, China

**Keywords:** permanent magnet synchronous motor, fault diagnosis, multi-sensor fusion, successive variational mode decomposition, spatiotemporal feature extraction, bidirectional LSTM, multi-head attention

## Abstract

With the increasing application of inverter-fed permanent magnet synchronous, motors (PMSMs) in industrial and intelligent energy systems, reliable fault detection and diagnosis (FDD) has become increasingly important for ensuring operational safety and system reliability. However, conventional single-sensor-based approaches usually exhibit limited robustness under varying operating conditions due to measurement noise, load fluctuations, and incomplete fault information. Therefore, multi-sensor information fusion has attracted increasing attention in PMSM fault diagnosis because it can provide complementary information from different sensing sources. This paper proposes a hybrid deep learning framework for multi-class PMSM fault diagnosis, integrating successive variational mode decomposition (SVMD)-based signal denoising, parallel temporal and spatial feature extraction using temporal convolutional network (TCN) and convolutional neural network (CNN), and BiLSTM with attention-based feature enhancement. First, SVMD is employed to adaptively decompose multi-sensor signals, and components with low Pearson correlation coefficients are removed as noise-dominated components. The remaining components are reconstructed to obtain denoised signals with improved quality. Subsequently, parallel TCN and CNN branches are constructed to extract temporal and spatial features, respectively, enabling comprehensive representation of spatiotemporal characteristics from multi-channel signals. Finally, a BiLSTM combined with an attention mechanism is utilized to model long-term dependencies and emphasize discriminative features for accurate fault classification. The proposed method is evaluated on a public PMSM dataset containing eight sensor channels and nine operating states. Experimental results demonstrate that the proposed framework achieves an accuracy of 98.5%, outperforming several existing representative models.

## 1. Introduction

Permanent magnet synchronous motors (PMSMs) are characterized by high power density, high efficiency, excellent dynamic performance, and a compact structure compared with induction motors and reluctance motors [1]. With the rapid development of renewable energy and intelligent power systems, PMSMs have been increasingly applied in various energy conversion fields, including nuclear power generation, wind power generation, and solar thermal power systems. In recent years, driven by advances in artificial intelligence and robotics technologies, PMSMs have also been widely adopted in intelligent robotic systems, such as industrial robots and humanoid robots, where high torque density, fast dynamic response, and precise motion control are critical for achieving reliable operation [2,3]. The increasing application of PMSM-driven robotic systems has further raised the requirements for motor reliability and fault diagnosis capability. However, similar to other electrical equipment, PMSMs may experience harsh operating conditions during long-term operation, including moisture, contamination, mechanical vibration, thermal stress, and electrical stress, which can accelerate degradation and increase the probability of faults [4]. PMSM faults can generally be categorized into motor-side faults, such as inter-turn short circuits and permanent magnet demagnetization, and inverter-related faults, including open-circuit faults, short-circuit faults, and overheating faults [5,6,7]. Therefore, accurate and timely detection of abnormal operating conditions is essential for ensuring reliable PMSM operation. Such detection strategies can support predictive maintenance, reduce unexpected shutdowns, and improve operational safety.

Existing PMSM fault diagnosis methods can mainly be classified into three categories: model-based methods, signal-based methods, and artificial intelligence-based methods. Model-based methods establish mathematical models of PMSMs under healthy and faulty conditions and perform diagnosis by analyzing residual differences between model outputs and measured signals [8,9,10]. These methods provide strong physical interpretability, require limited historical data, and maintain good robustness under varying operating conditions. However, several limitations restrict their practical applications. First, diagnostic performance strongly depends on model accuracy. Model uncertainties and insufficient representation of nonlinear system dynamics may result in false alarms or missed detections [11]. Moreover, increasing system complexity and coupling effects further complicate model construction, limiting their applicability in practical engineering scenarios.

Signal-based diagnosis methods identify PMSM faults by extracting discriminative features from measured sensor signals, such as current, voltage, vibration, and temperature signals [12,13]. According to the analysis domain, these methods can generally be classified into time-domain, frequency-domain, and time-frequency-domain approaches. Time-domain methods characterize signal variations using statistical features, such as peak value and mean value, and provide intuitive representations of different operating conditions [14]. However, their diagnostic performance is often affected by measurement noise and their limited sensitivity to weak early-stage faults [15]. Frequency-domain methods extract fault-related frequency components through spectral analysis techniques, such as Fourier transform. Although these methods are computationally efficient and easy to implement, they may lose temporal information and show limited capability in analyzing non-stationary signals and transient fault characteristics [16].

To overcome the limitations of time-domain and frequency-domain methods, time-frequency analysis approaches have been widely adopted for PMSM fault diagnosis because they can simultaneously characterize signal variations in both temporal and spectral domains. The short-time Fourier transform (STFT) provides time-frequency representations but suffers from the trade-off between time and frequency resolutions caused by fixed window sizes [17]. The continuous wavelet transform (CWT) improves adaptability through variable-scale analysis; however, its capability in extracting high-frequency components remains limited [16].

Adaptive signal decomposition methods have attracted considerable attention for nonlinear and non-stationary signal analysis. Empirical mode decomposition (EMD) decomposes complex signals into intrinsic mode functions (IMFs) without predefined basis functions, but it suffers from mode mixing when signal components have similar frequency characteristics [14]. To improve decomposition stability, ensemble empirical mode decomposition (EEMD) and complete ensemble empirical mode decomposition with adaptive noise (CEEMDAN) were subsequently developed by introducing noise-assisted strategies, which effectively reduce mode mixing and improve noise robustness [18,19]. However, these methods still involve additional computational costs and may be affected by residual noise. To further enhance decomposition performance, variational mode decomposition (VMD) was proposed and demonstrated improved noise robustness and reduced mode mixing.

Recently, several adaptive decomposition approaches have been developed for complex industrial signals. For example, adaptive variational empirical mode decomposition (AVEMD) introduces adaptive parameter adjustment strategies to improve decomposition performance under noisy and time-varying conditions [20]. However, such methods may require additional optimization procedures, increasing computational complexity and parameter dependence. Successive variational mode decomposition (SVMD), as an improved version of VMD, extracts signal modes sequentially without presetting the number of modes, providing better adaptability for complex signal decomposition [21]. Therefore, SVMD is adopted in this study to enhance the quality of multi-sensor signals before subsequent fault feature learning.

Unlike model-based and signal-based approaches, artificial intelligence (AI)-based fault diagnosis methods do not require explicit mathematical models or manually designed features. Instead, they automatically learn nonlinear relationships and discriminative patterns from collected sensor data, making them suitable for complex PMSM fault diagnosis tasks [22]. Existing AI methods can generally be categorized into shallow learning models, deep learning models, and hybrid models. Shallow learning algorithms, including support vector machines (SVMs), k-nearest neighbors (KNN), random forests, and multilayer perceptrons (MLPs), have achieved promising results in PMSM fault diagnosis. However, their limited feature representation capability makes them less effective when dealing with high-dimensional and complex sensor data [23,24].

Deep learning methods have demonstrated superior feature extraction capability by automatically learning hierarchical representations from raw signals. Among them, convolutional neural networks (CNNs) and long short-term memory networks (LSTMs) are two widely adopted architectures for PMSM fault diagnosis. CNNs can effectively extract spatial and local feature patterns from sensor signals through convolution operations, but their ability to capture long-term temporal dependencies is limited [25]. In contrast, LSTMs are capable of modeling sequential characteristics and temporal evolution of fault signals, while their ability to capture spatial correlations among multiple sensors remains insufficient. Therefore, hybrid architectures that combine CNNs and LSTMs have attracted increasing attention by integrating complementary spatial and temporal feature extraction capabilities.

Several studies have demonstrated the effectiveness of hybrid deep learning models for PMSM fault diagnosis. Tang et al. [25] developed a DW-CNN-LSTM framework that extracted multi-scale features from current signals and improved anomaly detection performance. Yu et al. [26] combined VMD with CNN-BiLSTM to identify inter-turn short-circuit and demagnetization faults, achieving robust classification performance. Meral [27] proposed a 1D-CNN-BiGRU model and verified its effectiveness on datasets containing inverter and stator winding faults. Furthermore, Yılmaz [28] developed a CNN-BiLSTM-attention framework for inverter-driven PMSM fault diagnosis and achieved satisfactory results on a public multi-sensor dataset. These studies demonstrate that deep learning-based hybrid models provide strong feature learning capability and have significant potential for PMSM condition monitoring.

Recently, temporal convolutional networks (TCNs) have attracted increasing attention due to their ability to capture long-range temporal dependencies through dilated causal convolutions while maintaining efficient parallel computation. Compared with traditional recurrent networks, TCNs can effectively model complex time-series characteristics and provide an alternative approach for temporal feature extraction. Therefore, combining TCNs with CNNs provides a promising strategy for simultaneously extracting temporal and spatial information from multi-channel PMSM signals.

Although existing AI-based methods have achieved considerable diagnostic accuracy, most studies still rely on single-sensor signals, such as current or vibration measurements [29]. However, single-sensor-based approaches may suffer from insufficient information representation and reduced robustness under complex operating conditions [30]. In contrast, multi-sensor systems can provide complementary information from different physical perspectives, thereby improving fault feature representation and diagnostic reliability [31,32]. Therefore, developing effective methods for multi-sensor information fusion and comprehensive feature learning is of great significance for advanced PMSM fault diagnosis.

To address the limitations of existing PMSM fault diagnosis methods, this paper proposes a hybrid deep learning framework integrating SVMD-based denoising, parallel CNN-TCN spatiotemporal feature extraction, and BiLSTM with attention-based feature enhancement. The proposed method is evaluated on a public PMSM dataset containing eight sensor channels and nine operating states. In this framework, SVMD is employed to improve input signal quality by reducing noise interference, while the parallel CNN-TCN module extracts complementary spatial and temporal features from multi-sensor signals. Subsequently, the BiLSTM-Attention module enhances fault-related feature representations by adaptively emphasizing important temporal information. Different from existing hybrid models that mainly combine multiple neural network modules to improve classification performance, the proposed framework is designed by considering the characteristics of PMSM multi-sensor fault signals, where different modules are assigned specific roles in signal enhancement and feature extraction.

The main contributions of this study are summarized as follows:

(1) A hybrid deep learning framework is proposed for multi-sensor and multi-class PMSM fault diagnosis. Different from existing hybrid models that mainly concatenate multiple neural networks, the proposed framework integrates signal enhancement, spatiotemporal feature extraction, and feature refinement modules according to the characteristics of PMSM fault signals.

(2) An SVMD-based adaptive denoising strategy is introduced to improve the quality of multi-sensor input signals. By decomposing sensor measurements into IMFs and removing weakly correlated noise components, the proposed preprocessing method reduces the influence of signal disturbances while preserving fault-related information.

(3) A parallel CNN-TCN feature extraction module is designed to simultaneously capture spatial correlations among multiple sensors and temporal dependencies within fault signals. This design enables more comprehensive feature representation compared with single-branch feature extraction methods.

The remainder of this paper is organized as follows. Section 2 introduces the theoretical background of the algorithms used in the proposed framework. Section 3 presents the architecture and implementation details of the proposed PMSM fault diagnosis method. Section 4 describes the experimental dataset and evaluation results, including comparisons with different models. Finally, Section 5 concludes this study and discusses future research directions.

## 2. Theoretical Background of the Proposed Framework

### 2.1. Successive Variational Mode Decomposition Algorithm

SVMD proposed by Nazari et al. in 2020, is an adaptive signal decomposition technique derived from VMD [33]. Compared with conventional VMD, SVMD eliminates the requirement of manually determining the number of decomposition modes in advance. Through variational optimization, SVMD decomposes the target signal into multiple band-limited modes with specific center frequencies [34,35].

The decomposition process can be expressed as:

(1) $$f(t)=\sum_{i=1}^{L}u_{i}(t)+r(t)$$

where $u_{i}(t)$ denotes the i-th extracted mode, L represents the number of obtained modes, and r(t) is the residual component. By minimizing the bandwidth of each extracted mode and reducing spectral overlap between adjacent modes, SVMD achieves adaptive signal decomposition with improved noise robustness.

In this study, SVMD is adopted as a preprocessing method for high-frequency sensor signals in PMSM fault diagnosis. The decomposed components are evaluated according to their correlation with the original signal, and components with low Pearson correlation coefficients are removed. The remaining components are reconstructed to enhance signal quality before subsequent feature extraction.

### 2.2. Convolutional Neural Networks

CNNs are deep learning architectures with strong feature extraction capabilities and have been widely applied in various fields, including fault diagnosis and pattern recognition [36]. The basic structure of CNNs mainly consists of convolutional layers and pooling layers. The convolutional layer extracts local feature representations from input data through learnable convolution kernels, which enables automatic feature extraction and hierarchical feature representation.

**Figure 1:**
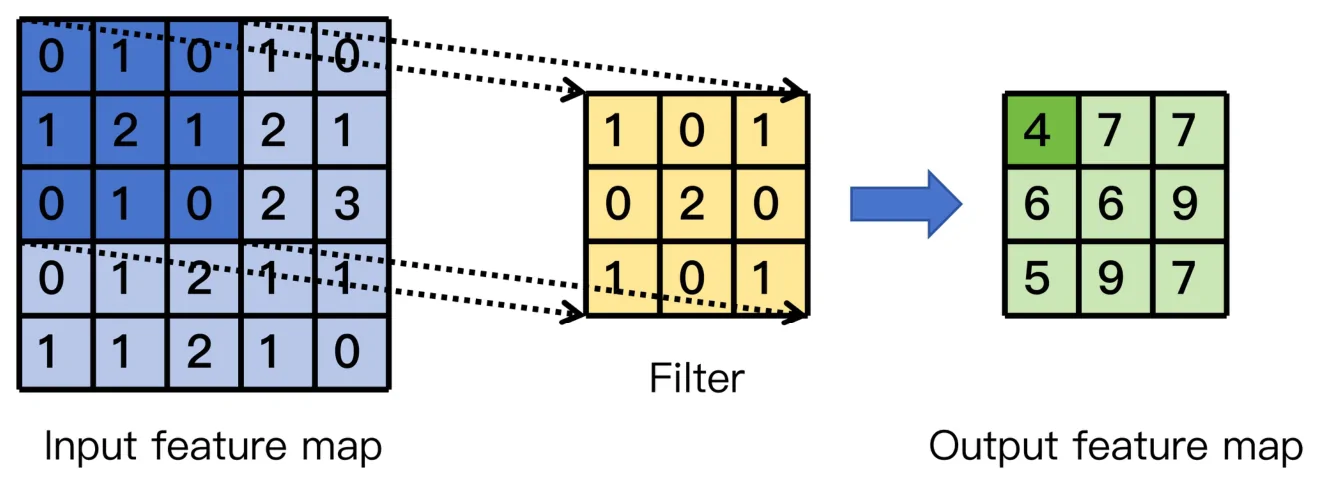
Convolution operation process.

The convolution operation has two important characteristics: parameter sharing and local connectivity. Parameter sharing allows the same convolution kernel to be applied across different positions of the input data, thereby reducing the number of trainable parameters and improving computational efficiency. Local connectivity enables the network to capture local feature patterns effectively, which enhances its capability to extract spatial correlations from complex input signals. The convolution operation process is illustrated in Figure 1.

**Figure 2:**
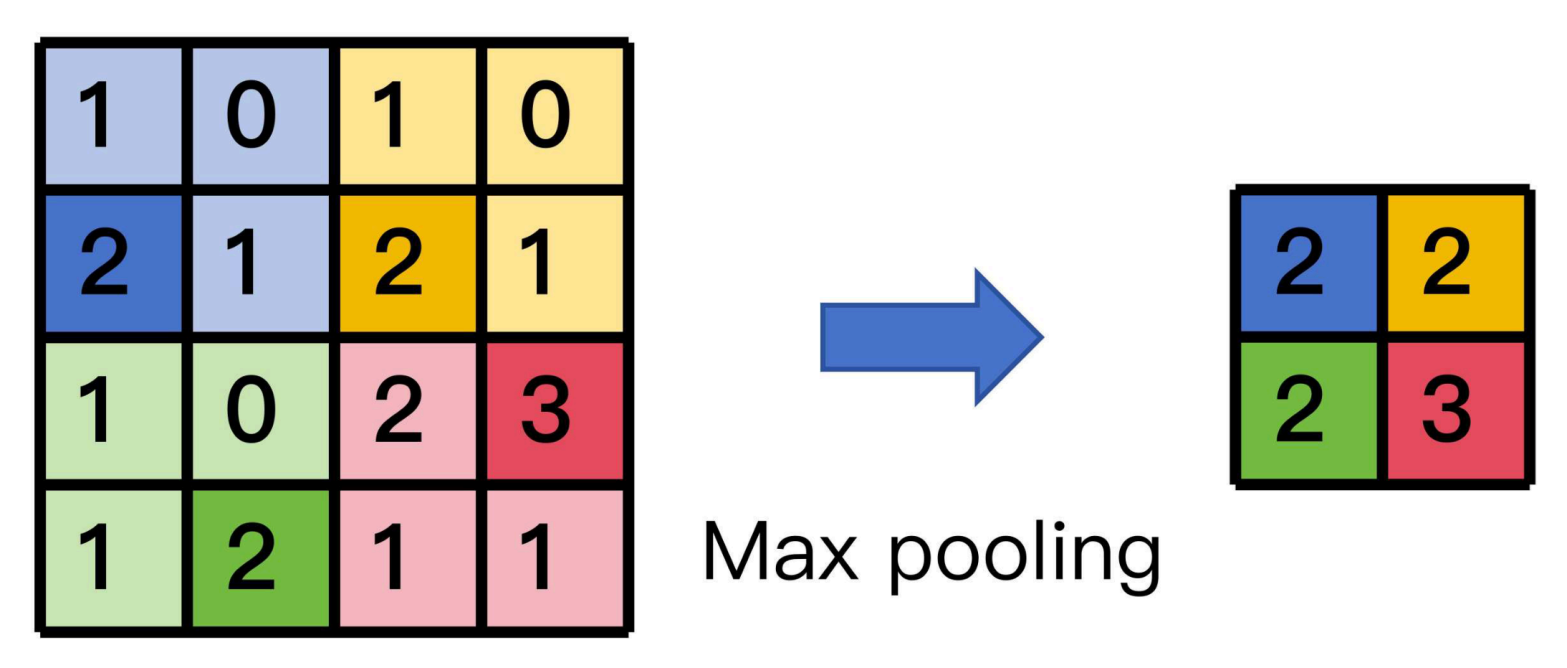
Max pooling operation process.

The pooling layer is mainly used to reduce the dimensionality of feature maps generated by convolution operations. By decreasing the spatial size of feature representations, pooling layers reduce computational complexity while preserving dominant features, thereby improving the robustness and generalization capability of CNNs [37]. Common pooling strategies include max pooling and average pooling. Max pooling selects the maximum value within each pooling region and retains the most representative features, whereas average pooling calculates the average value of the region. In this study, max pooling is adopted to preserve prominent fault-related features, and its operation principle is illustrated in Figure 2.

### 2.3. Temporal Convolutional Network

TCN was proposed by Bai et al. in 2018 as a deep learning architecture for sequence modeling [38,39]. Compared with traditional recurrent neural networks, TCN can capture long-range temporal dependencies while maintaining parallel computation efficiency. By combining causal convolution, dilated convolution, and residual connections, TCN effectively enlarges the receptive field and extracts temporal patterns from sequential data.

Causal convolution is a fundamental component of TCN that ensures the output at the current time step only depends on the current and previous inputs, thereby preventing the use of future information during temporal feature extraction. The mathematical formulation of causal convolution can be expressed as:

(2) $$y_{t}=\sum_{i=0}^{k-1}w_{i}x_{t-i}$$

where $x_{t-i}$ and $y_{t}$ represent the input and output at different time steps, respectively; k denotes the convolution kernel size, and $w_{i}$ represents the corresponding kernel weight.

**Figure 3:**
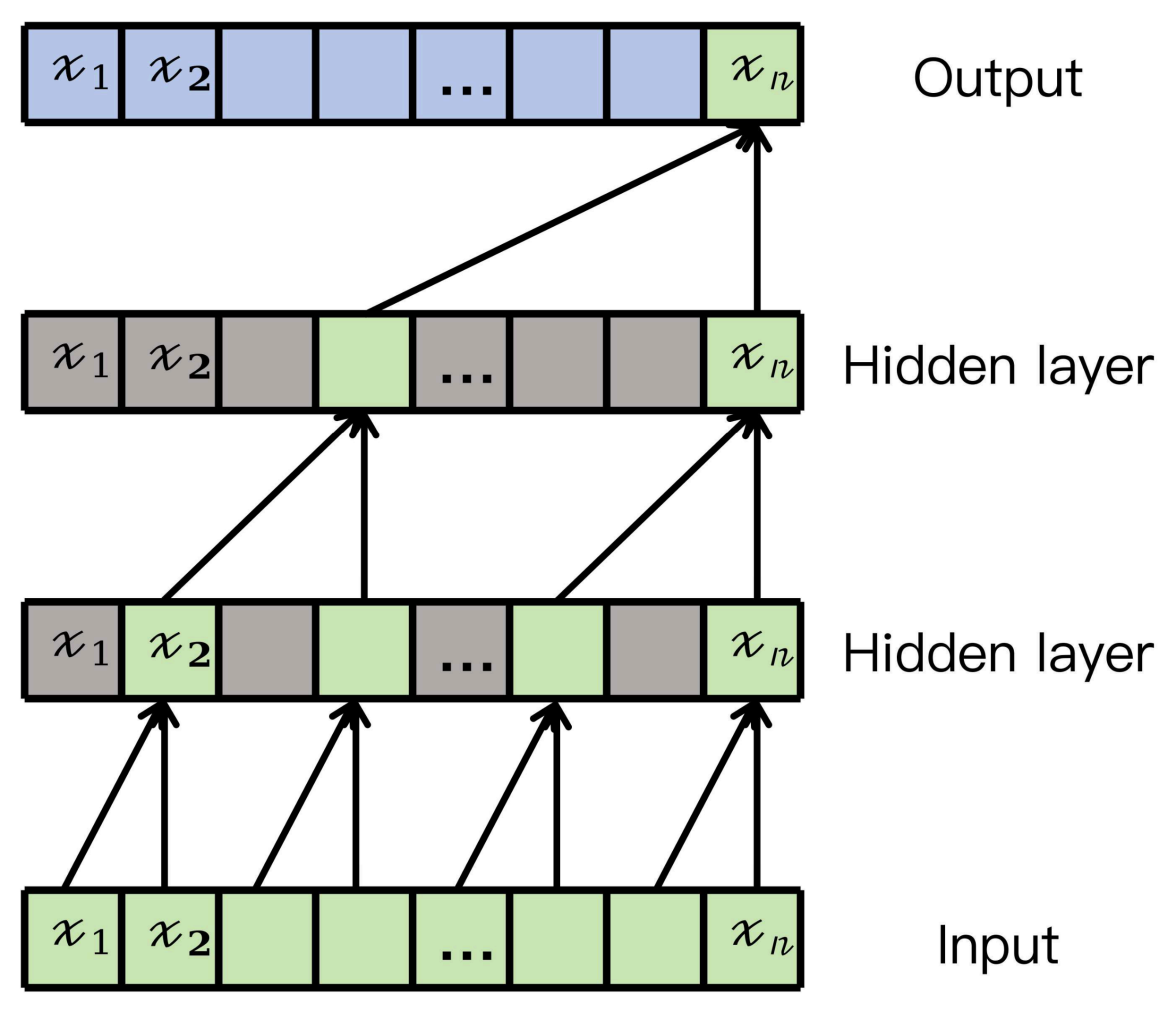
Principle of dilated convolution.

To further expand the receptive field without significantly increasing network parameters, TCN introduces dilated convolution. By inserting fixed intervals between convolution kernel elements, dilated convolution enables the network to capture long-range temporal dependencies while maintaining computational efficiency. The operation principle of dilated convolution is illustrated in Figure 3, and its mathematical expression is given as:

(3) $$y_{t}=\sum_{i=0}^{k-1}w_{i}x_{t-di}$$

where d represents the dilation factor that determines the interval between sampled input elements.

In addition, TCN employs residual connections to improve network optimization. The residual structure introduces a skip connection that directly transfers the input to the output, allowing the network to learn residual mappings instead of complete transformations. This design alleviates gradient vanishing and degradation problems in deep networks and improves training stability. The residual operation can be expressed as:

(4) H(X)=F(X)+X

where X denotes the input feature, F(X) represents the nonlinear transformation performed by convolutional layers, and H(X) is the final output of the residual block.

### 2.4. Bidirectional Long Short-Term Memory Network

BiLSTM is an extension of the conventional LSTM architecture that consists of two independent LSTM layers operating in the forward and backward directions [40]. By processing sequential data from both temporal directions, BiLSTM can capture contextual information from different temporal perspectives. At each time step, the hidden states generated by the forward and backward LSTM layers are combined to form a comprehensive feature representation, enabling the model to effectively learn long-term dependencies in sequential signals.

**Figure 4:**
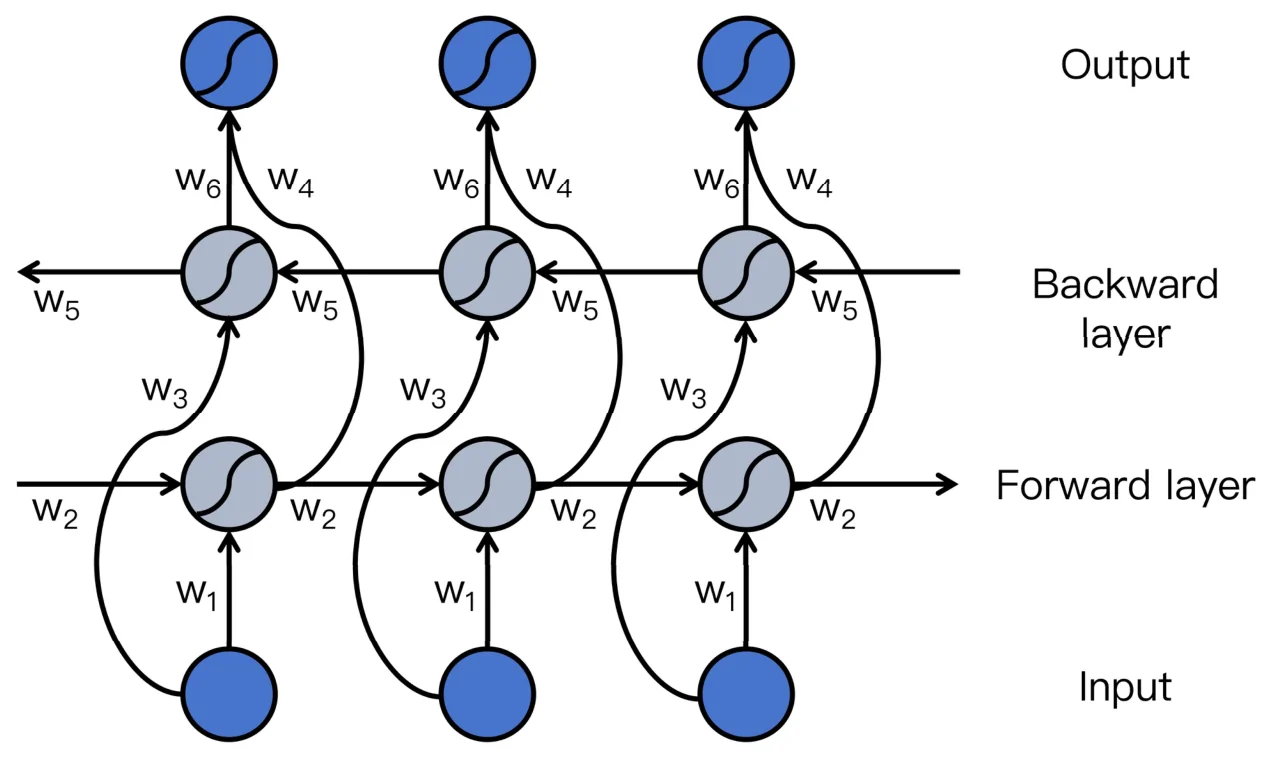
Structure of BiLSTM.

Compared with traditional unidirectional LSTM networks, BiLSTM provides richer temporal representations and has stronger capability in modeling complex sequence patterns. Therefore, it has been widely applied in time-series analysis and fault diagnosis tasks. In this study, BiLSTM is employed to further learn long-range dependencies from the extracted spatiotemporal features and enhance the discriminative capability of fault representations. The structure of BiLSTM is illustrated in Figure 4.

### 2.5. Multi-Head Attention

The attention mechanisms are effective feature enhancement approachs that adaptively assign different weights to input features according to their relevance. By modeling the relationships among different feature representations, attention mechanisms enable neural networks to focus on more informative features while reducing the influence of irrelevant information [41].

The scaled dot-product attention mechanism maps the input features into three vectors: query (Q), key (K), and value (V). The relevance between Query and Key is calculated through the dot-product operation, and the weighted feature representation is obtained by applying the attention weights to Value. The mathematical formulation is expressed as:

(5) $$Attention(Q,K,V)=softmax(\frac{QK^{T}}{\sqrt{d_{k}}})V$$

where $d_{k}$ denotes the dimension of the Key vector.

To enhance the feature learning capability, the multi-head attention mechanism performs multiple independent attention operations in different representation subspaces. Specifically, query, key, and value are linearly projected into multiple subspaces, where each projection generates an individual attention head. This design enables the model to capture diverse feature correlations from different representation perspectives [42]. The calculation process of each attention head is given as:

(6) $${head}_{i}=Attention(QW_{i}^{Q},KW_{i}^{K},VW_{i}^{V})$$

The outputs of all attention heads are concatenated and linearly transformed to obtain the final attention representation:

(7) $$Multihead(Q,K,V)=concat({head}_{1},{head}_{2},\cdots ,{head}_{h})W^{O}$$

where $W_{i}^{Q}$, $W_{i}^{K}$, $W_{i}^{V}$, and $W^{O}$ are learnable weight matrices.

In this study, the multi-head attention mechanism is integrated with BiLSTM to adaptively emphasize fault-related temporal features and improve the discriminative representation capability of the extracted spatiotemporal features.

## 3. The Proposed Fault Diagnosis Methodology

### 3.1. Dataset Description

**Figure 5:**
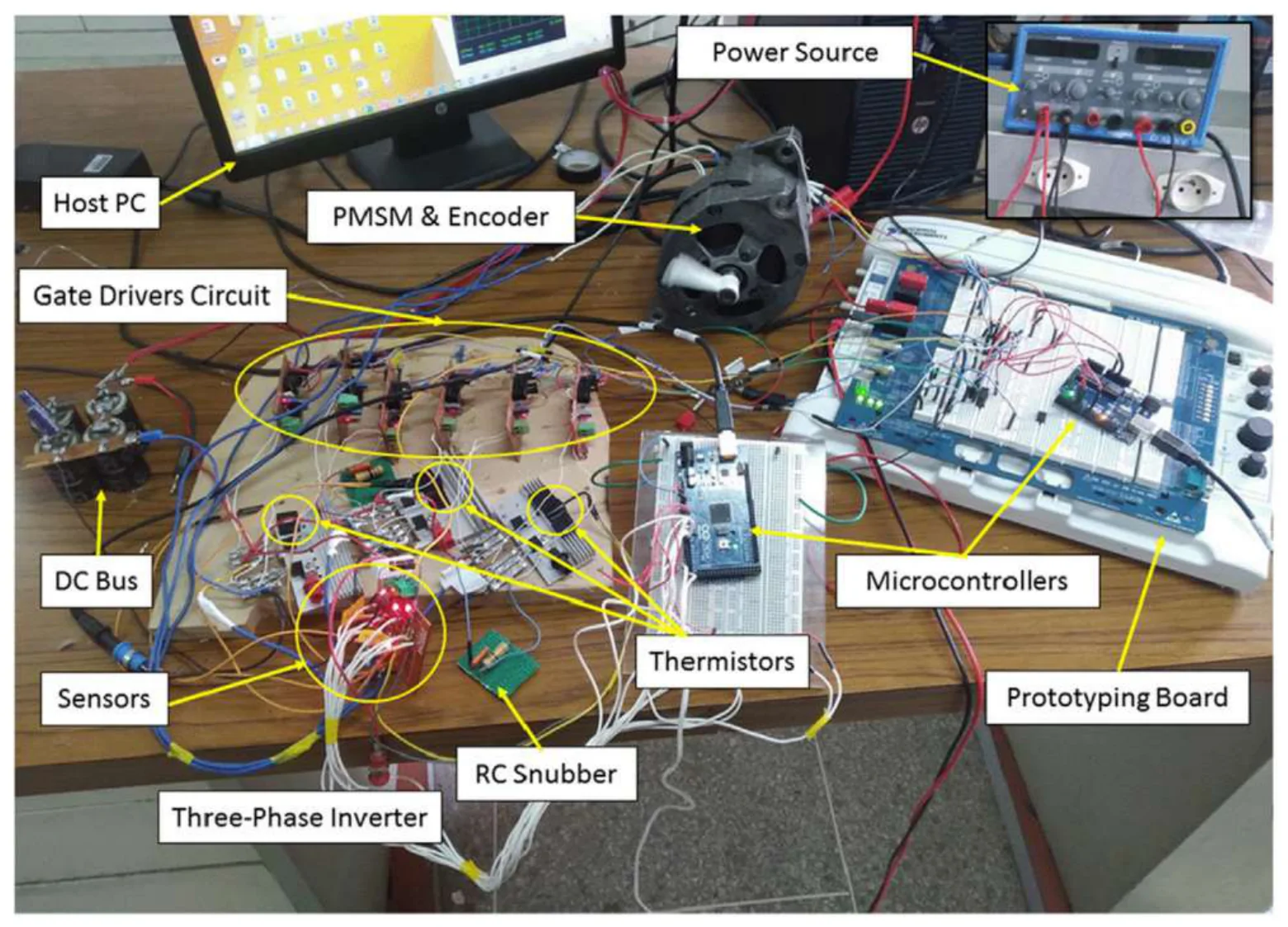
Experimental platform for PMSM data acquisition [43].

The experimental evaluation in this study is based on the public PMSM fault diagnosis dataset released by Bacha et al. [43], which was developed for fault detection and diagnosis (FDD) of inverter-fed PMSM systems. The dataset was collected using a dedicated PMSM experimental platform, as illustrated in Figure 5. The platform consists of a PMSM, an inverter, a DC power supply, multiple sensors, and an Arduino-based data acquisition system. The experimental platform and data acquisition process were established by the dataset providers; therefore, the present study uses the collected dataset for algorithm evaluation rather than conducting experiments on a self-built PMSM prototype.

During data acquisition, the motor operated at a speed of 10 rad/s with a DC supply voltage of 15 V under an ambient temperature of 25 °C. The sampling frequency was 10 Hz, resulting in a total of 10,892 samples. Current and voltage signals were measured using ACS712 20 A Hall-effect sensors together with 100 Ω series resistors, whereas temperature was acquired using a 10 kΩ NTC thermistor.

The dataset includes one normal operating condition (F0) and eight fault conditions (Table 1), covering open-circuit faults (F1 and F2), short-circuit faults (F3–F5), and overheating faults affecting individual or multiple half-bridge modules (F6–F8). The distribution of each fault category is summarized in Table 1. Each sample consists of eight raw sensor measurements and fifteen derived features. The raw measurements include two phase currents (Ia and Ib), DC bus voltage (VDC), DC bus current (IDC), three half-bridge temperatures (T1, T2, and T3), and driver voltage (VD). The derived features describe additional electrical and thermal characteristics, including physical unit conversions, DC and AC power, current imbalance, maximum temperature difference, normalized currents, as well as moving averages and rates of change of key signals. Following the feature selection strategy in [28], seven redundant derived features are removed, while all eight raw sensor measurements and ten representative derived features are retained for subsequent analysis. The selected derived features include Ia_original, Ib_original, Power_DC, Power_AC, Current_Imbalance, Temp_Diff_Max, VDC_RateOfChange, IDC_RateOfChange, VDC_MovingAvg, and IDC_MovingAvg. Detailed descriptions of all retained features are provided in Table 2.

It should be noted that the dataset was collected under a fixed motor speed, DC supply voltage, and ambient temperature. Therefore, the experimental results in this study are intended to evaluate the fault diagnosis capability of the proposed method under the operating condition covered by the dataset and do not fully establish its generalization to varying operating conditions. Further validation using PMSM data collected under different speeds, voltages, temperatures, and load conditions is required to assess the cross-condition generalization of the proposed method.

### 3.2. Data Preprocessing

To prevent data leakage caused by highly correlated consecutive samples, the dataset is first divided according to fault categories before sample construction. Specifically, the data corresponding to each fault type are randomly split into training, validation, and test subsets with proportions of 65%, 20%, and 15%, respectively. The subsets from all fault categories are subsequently merged to form the final training, validation, and test sets while preserving the class distribution.

After dataset partitioning, samples are generated using a sliding-window strategy with a window size of 15 data points and a stride of 5. The window size is selected according to previous studies on the same PMSM fault diagnosis dataset, where different window lengths were evaluated and a window size of 15 achieved a favorable balance between temporal information preservation and diagnostic performance [28]. For the adopted PMSM operating signals, this window length is sufficient to capture short-term dynamic characteristics while maintaining an appropriate sample size. A stride of 5 is employed to preserve temporal continuity between adjacent samples. Based on these settings, 1413 training samples, 432 validation samples, and 324 test samples are generated.

Finally, feature standardization is performed using the StandardScaler implementation in scikit-learn (version 1.9.0). The mean and standard deviation are calculated exclusively from the training set, and the same transformation is subsequently applied to the validation and test sets to eliminate information leakage and ensure a fair evaluation of the proposed framework.

### 3.3. Denoising Processing Based on SVMD

The raw sensor signals inevitably contain noise due to measurement uncertainty, environmental interference, and hardware limitations. These disturbances may obscure fault-related characteristics, making feature extraction more challenging and consequently degrading the performance of fault diagnosis models. For nonlinear and non-stationary industrial signals, adaptive decomposition methods have been widely used for noise reduction and feature enhancement. However, some methods, such as EEMD and CEEMDAN, require repeated decomposition processes, resulting in increased computational costs, while adaptive parameter optimization methods may introduce additional parameter dependence. Therefore, considering the multi-sensor characteristics and noise distribution differences among PMSM sensor signals, SVMD is adopted in this study as the preprocessing method due to its sequential decomposition mechanism and capability of extract informative signal components without requiring prior determination of the number of modes.

**Figure 6:**
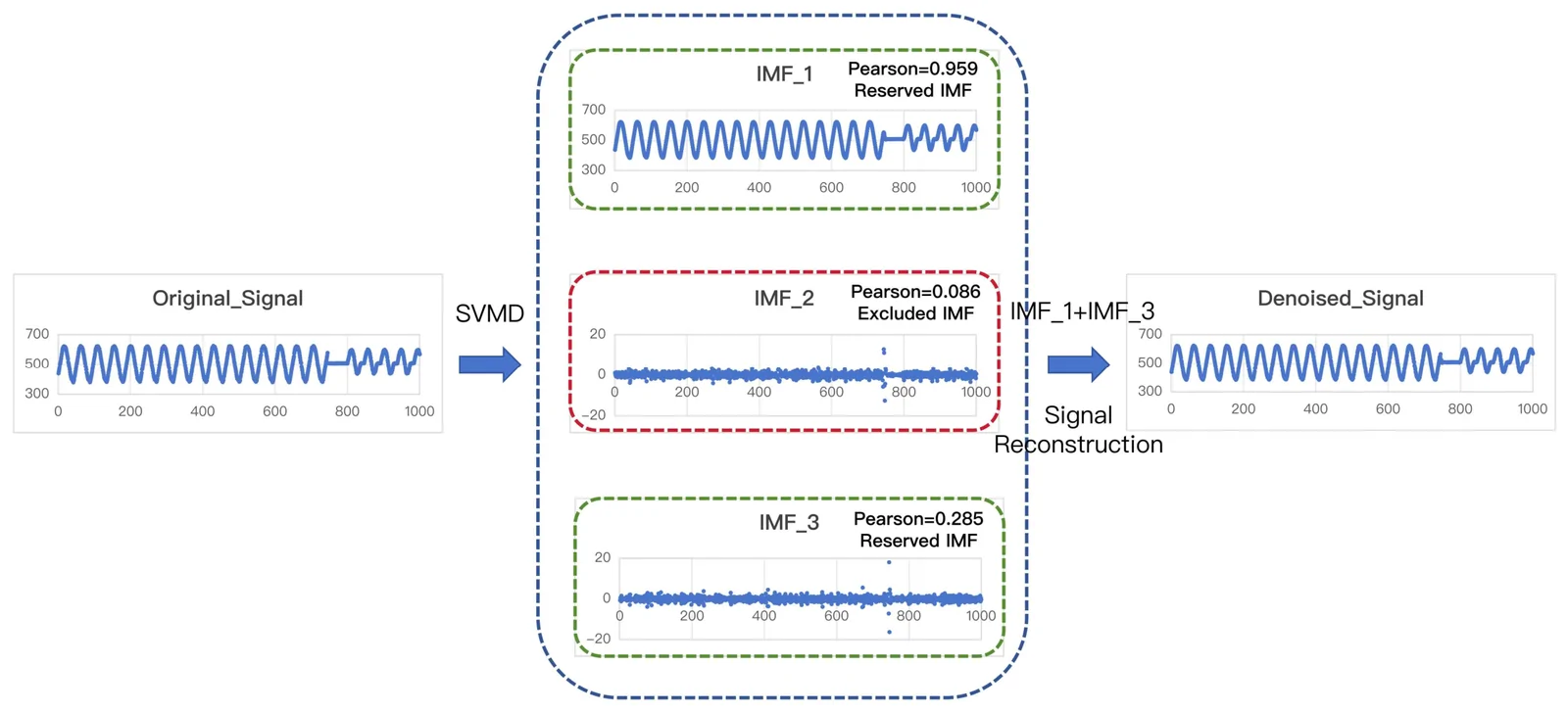
Workflow of the proposed SVMD-based denoising process.

In this study, SVMD is employed to denoise each feature signal, and the overall procedure is illustrated in Figure 6. First, each feature signal is decomposed into multiple IMF components using SVMD. Next, the Pearson correlation coefficient between each IMF component and the corresponding original signal is calculated to evaluate their relevance. IMFs with correlation coefficients lower than 0.1 are regarded as noise-dominated components and are discarded. Finally, the remaining IMF components are reconstructed to generate the denoised signal, which is subsequently used for model training and fault diagnosis.

SVMD is applied to all 18 input features, including eight raw sensor measurements and ten derived features. For each feature, the Pearson correlation coefficient between every IMF component and the corresponding original signal is calculated to identify noise-dominated components. As summarized in Table 3, several current-related and temperature-related features contain IMF components with correlation coefficients below 0.1, indicating the presence of significant noise. These low-correlation components are removed during signal reconstruction. In contrast, all IMF components obtained from the remaining features exhibit correlation coefficients greater than 0.1, suggesting that no obvious noise components are present; therefore, these features are used directly without reconstruction.

In this study, a Pearson correlation threshold of 0.1 is adopted for IMF component selection. This threshold is determined according to the distribution characteristics of the calculated correlation coefficients. As shown in Table 3, the first IMF components exhibit high correlations with their corresponding original signals (0.958–0.999), indicating that they preserve the dominant characteristics of the measured signals. In contrast, several higher-order IMF components show correlation coefficients lower than 0.1, suggesting that these components mainly contain noise-related information.

To further verify the rationality of the selected threshold, a sensitivity analysis is conducted by evaluating different Pearson correlation thresholds. The corresponding diagnosis results are summarized in Table 4. The results show that the threshold of 0.1 achieves the best performance, with both the Accuracy and F1-score reaching 0.985. When no IMF filtering is applied, the accuracy decreases to 0.972, indicating that retaining all decomposed components introduces redundant noise information. When the threshold is increased to 0.2, the performance decreases significantly because some weakly correlated but potentially useful fault-related components may also be removed. The threshold of 0.08 achieves comparable performance, but it does not provide the best balance between noise suppression and fault feature preservation. Therefore, the Pearson correlation threshold of 0.1 is selected in this study as a suitable compromise between denoising effectiveness and fault information retention.

### 3.4. Feature Extraction and Fault Detection Model

**Figure 7:**
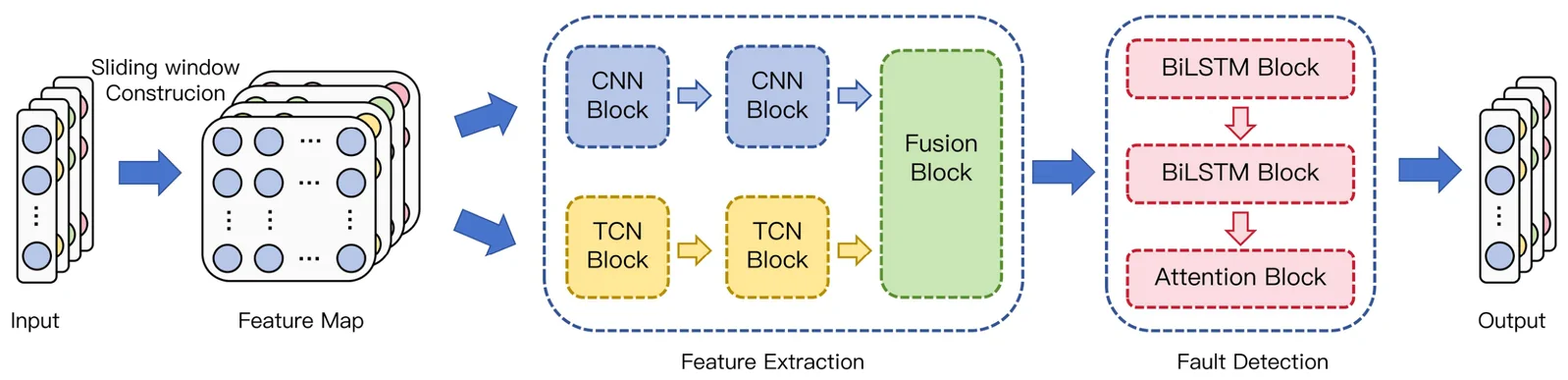
Architecture of the proposed hybrid fault diagnosis model.

The architecture of the proposed hybrid fault diagnosis model is illustrated in Figure 7, and the detailed configurations of each layer are summarized in Table 5. The proposed framework integrates a parallel CNN-TCN module for spatiotemporal feature extraction and a BiLSTM–Attention module for discriminative feature enhancement. By exploiting complementary information from multiple sensors, the proposed framework aims to achieve accurate multi-class fault diagnosis of PMSMs.

After SVMD-based denoising and data preprocessing, the input samples with dimensions of 15 × 18 are simultaneously fed into the CNN and TCN branches. The CNN branch is designed to extract cross-sensor feature interactions and local patterns from multi-channel input sequences. In this study, the cross-sensor correlations refer to the dependencies among different sensor channels in the feature dimension rather than physical spatial relationships. Since different sensors measure complementary information related to PMSM operating conditions and fault characteristics, convolution operations can effectively learn local feature interactions among channels and extract discriminative fault-related representations. It consists of two one-dimensional convolutional layers with kernel sizes of 3. Each convolutional layer is followed by batch normalization, ReLU activation, and dropout regularization with a rate of 0.2. A max pooling operation is subsequently applied to reduce feature dimensionality and improve computational efficiency.

In parallel, the TCN branch is developed to model temporal dependencies within sensor signals. Two dilated one-dimensional convolutional layers are employed to enlarge the receptive field while preserving temporal information. The Chomp layer is introduced to remove redundant padding generated by causal convolutions, ensuring that the output sequence maintains the original temporal dimension. Furthermore, residual connections are adopted to facilitate gradient propagation and improve the capability of temporal feature learning.

The feature representations extracted from the CNN and TCN branches are considered complementary, where the CNN branch focuses on learning cross-channel feature interactions and the TCN branch captures temporal evolution patterns. The extracted spatial and temporal representations are then integrated through the Fusion block. Specifically, a one-dimensional convolution with a kernel size of 1 is utilized to perform channel-wise feature fusion, generating a unified spatiotemporal feature representation. The fused features are subsequently fed into the BiLSTM–Attention module for further feature enhancement.

The BiLSTM module is employed to capture long-term temporal dependencies within the fused feature sequences. It consists of two stacked BiLSTM layers, which simultaneously learn forward and backward temporal information within each input window. Compared with unidirectional sequence modeling, the bidirectional structure provides richer contextual information and improves the representation of fault-related temporal characteristics.

To further enhance the discriminative capability of the extracted features, a multi-head attention mechanism is introduced after the BiLSTM module. Four attention heads with a key dimension of 16 are employed to learn feature representations from different subspaces. The outputs of multiple attention heads are concatenated and fused, allowing the model to adaptively assign higher weights to informative temporal features while suppressing less relevant information.

Finally, the classifier head transforms the enhanced feature representation into fault classification results. Global average pooling is first applied to aggregate the attention output sequence into a fixed-length feature vector. The vector is then processed by two fully connected layers with ReLU activation and dropout regularization (0.2). The final dense layer with softmax activation outputs a nine-dimensional probability vector corresponding to the nine PMSM operating states.

## 4. Experimental Results and Analysis

### 4.1. Evaluation Metrics

To comprehensively evaluate the performance of different fault diagnosis models for PMSMs, four widely used classification metrics, namely accuracy, precision, recall, and F1-score, are adopted in this study. These metrics evaluate model performance from different perspectives and provide complementary information, enabling a comprehensive assessment of classification effectiveness. They are particularly suitable for multi-class fault diagnosis tasks with imbalanced class distributions.

Accuracy measures the overall proportion of correctly classified samples. Precision evaluates the reliability of positive predictions, while recall reflects the ability of the classifier to correctly identify samples belonging to each target class. The F1-score, defined as the harmonic mean of precision and recall, provides a balanced evaluation by jointly considering both metrics. The corresponding formulas are given as follows:

(8) $$Accuracy=\frac{TP+TN}{TP+TN+FP+FN}$$

(9) $$Precision=\frac{TP}{TP+FP}$$

(10) $$Recall=\frac{TP}{TP+FN}$$

(11) $$F1-Score=2\times \frac{Precision\times Recall}{Precision+Recall}$$

where TP, TN, FP and FN denote the numbers of true positives, true negatives, false positives, and false negatives, respectively.

### 4.2. Comparison with Baseline Models

To evaluate the effectiveness of the proposed framework, five representative baseline models are selected for comparison. The overall fault diagnosis performance of all models is summarized in Table 6. The comparison focuses on two aspects: (1) evaluating the contribution of SVMD-based signal denoising to fault diagnosis performance, and (2) investigating the effectiveness of the proposed parallel CNN-TCN architecture by comparing it with models employing only CNN- or TCN-based feature extraction.

As shown in Table 6, incorporating SVMD as a signal preprocessing strategy consistently improves the performance of all models. Compared with Models 4–6, the corresponding Models 1–3 with SVMD achieve higher accuracy, precision, recall, and F1-score, indicating that SVMD effectively suppresses noise interference and enhances the quality of the input signals. Consequently, more discriminative fault features can be extracted, leading to improved fault diagnosis performance.

A comparison between the CNN-based and TCN-based models further shows that the CNN branch achieves slightly better overall performance than the TCN branch. This result suggests that spatial correlations among multi-sensor signals provide more discriminative information than temporal dependencies for the adopted PMSM dataset. Nevertheless, the performance gap between the two architectures is relatively small, indicating that both spatial and temporal features contribute to fault identification.

By jointly exploiting these complementary characteristics, the proposed parallel CNN-TCN architecture achieves superior performance over models employing either CNN or TCN alone. The fused spatiotemporal representations enable the model to capture more comprehensive fault characteristics, thereby improving classification accuracy and overall diagnostic performance. Among all evaluated models, the proposed framework achieves the best overall performance, with accuracy, precision, recall, and F1-score all reaching 98.5%.

### 4.3. Per-Class Fault Identification Performance

To further evaluate the discriminative capability of different models for individual fault categories, the per-class classification accuracy is reported in Table 7. As shown in the table, all models achieve nearly perfect recognition accuracy for the overheating faults (F6–F8), indicating that these fault types exhibit highly distinguishable characteristics and can therefore be accurately separated from other operating conditions.

In contrast, relatively lower recognition accuracy is observed for the low-side short-circuit fault (F3) across all evaluated models. This phenomenon is mainly related to the limited number of samples available for this fault category. Specifically, only 12 F3 samples are included in the test set, accounting for a small proportion of the total 324 test samples. Therefore, the classification accuracy of F3 is highly sensitive to individual prediction errors, and a small number of misclassified samples can result in a considerable decrease in the calculated accuracy.

It is worth noting that the lower recognition accuracy of F3 is also observed in other comparison models, indicating that this fault category remains challenging to identify under the current dataset. In addition to the limited number of test samples, similarities in signal characteristics between F3 and other fault categories may also contribute to the misclassification. Further analysis of the specific feature differences among these fault types will be investigated in future work.

After introducing SVMD-based signal denoising, all models exhibit improved classification performance across different fault categories, with particularly noticeable improvements for the high-side open-circuit fault (F1) and the high-side short-circuit fault (F4) at S3. These results indicate that SVMD effectively enhances the discriminability of fault-related features by suppressing noise components in the original sensor signals.

For the normal operating condition (F0), both the CNN-based model and the proposed parallel CNN-TCN model demonstrate superior recognition performance. In particular, the CNN-based model achieves perfect classification for F0, suggesting that spatial feature learning is highly effective in characterizing the stable operating patterns of the PMSM under normal conditions. Overall, the proposed framework achieves the most balanced classification performance across different operating states, demonstrating its superior capability for multi-class PMSM fault diagnosis.

### 4.4. Confusion Matrix Analysis

**Figure 8:**
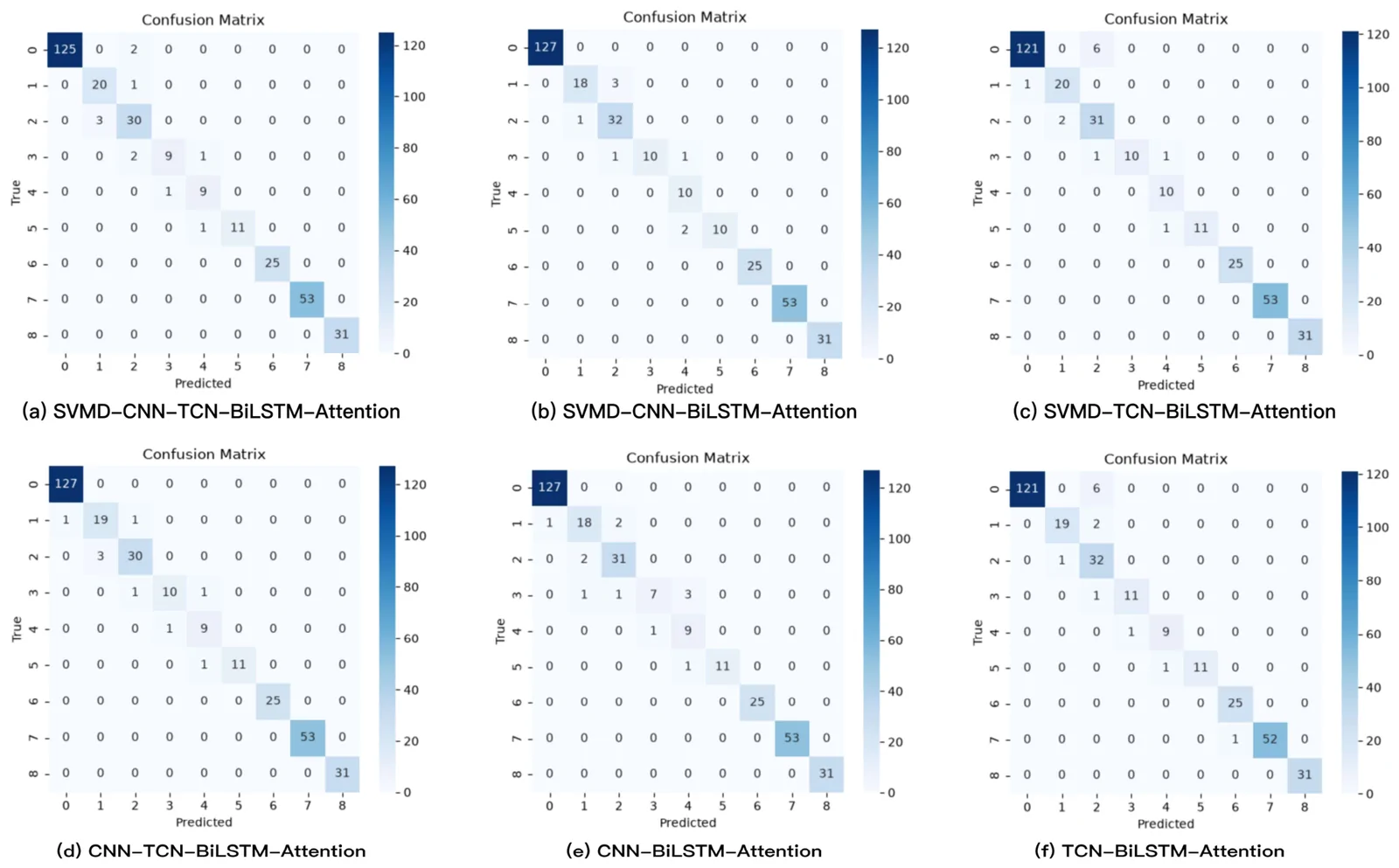
Confusion matrices of different fault diagnosis models.

Confusion matrices provide an intuitive visualization of the relationship between predicted labels and actual classes, enabling a detailed analysis of classification performance and misclassification patterns among different operating states. The confusion matrices of the evaluated models are presented in Figure 8.

As shown in Figure 8, the TCN-based temporal feature extraction model exhibits misclassification between the normal operating condition (F0) and the low-side open-circuit fault at position S6 (F2). Specifically, six normal samples are incorrectly classified as F2, resulting in a reduced recognition accuracy for the normal operating condition. This observation suggests that temporal representations alone may have limited discriminative capability when distinguishing operating states with similar signal variation patterns.

In comparison, the CNN-based spatial feature extraction model achieves 100% recognition accuracy for the normal operating condition (F0). This indicates that cross-sensor correlations among multi-sensor measurements provide complementary discriminative information for separating normal and faulty states. Furthermore, the proposed parallel CNN-TCN feature extraction strategy further improves the overall classification performance by jointly exploiting spatial and temporal characteristics. The fusion of these complementary features enables the model to better characterize the differences between operating states and reduces misclassification caused by relying on a single feature representation.

For fault-state recognition, the CNN-TCN model still exhibits some confusion between similar fault categories. In particular, the low-side open-circuit fault at S6 (F2) is occasionally misclassified as the high-side open-circuit fault at S3 (F1), indicating that these two fault conditions share partially similar feature distributions. Nevertheless, compared with models based on individual feature extraction strategies, the CNN-TCN framework achieves more balanced recognition performance across different fault categories. These results demonstrate the effectiveness of spatiotemporal feature fusion in enhancing the robustness and reliability of PMSM multi-class fault diagnosis.

### 4.5. Feature Visualization Based on t-SNE

**Figure 9:**
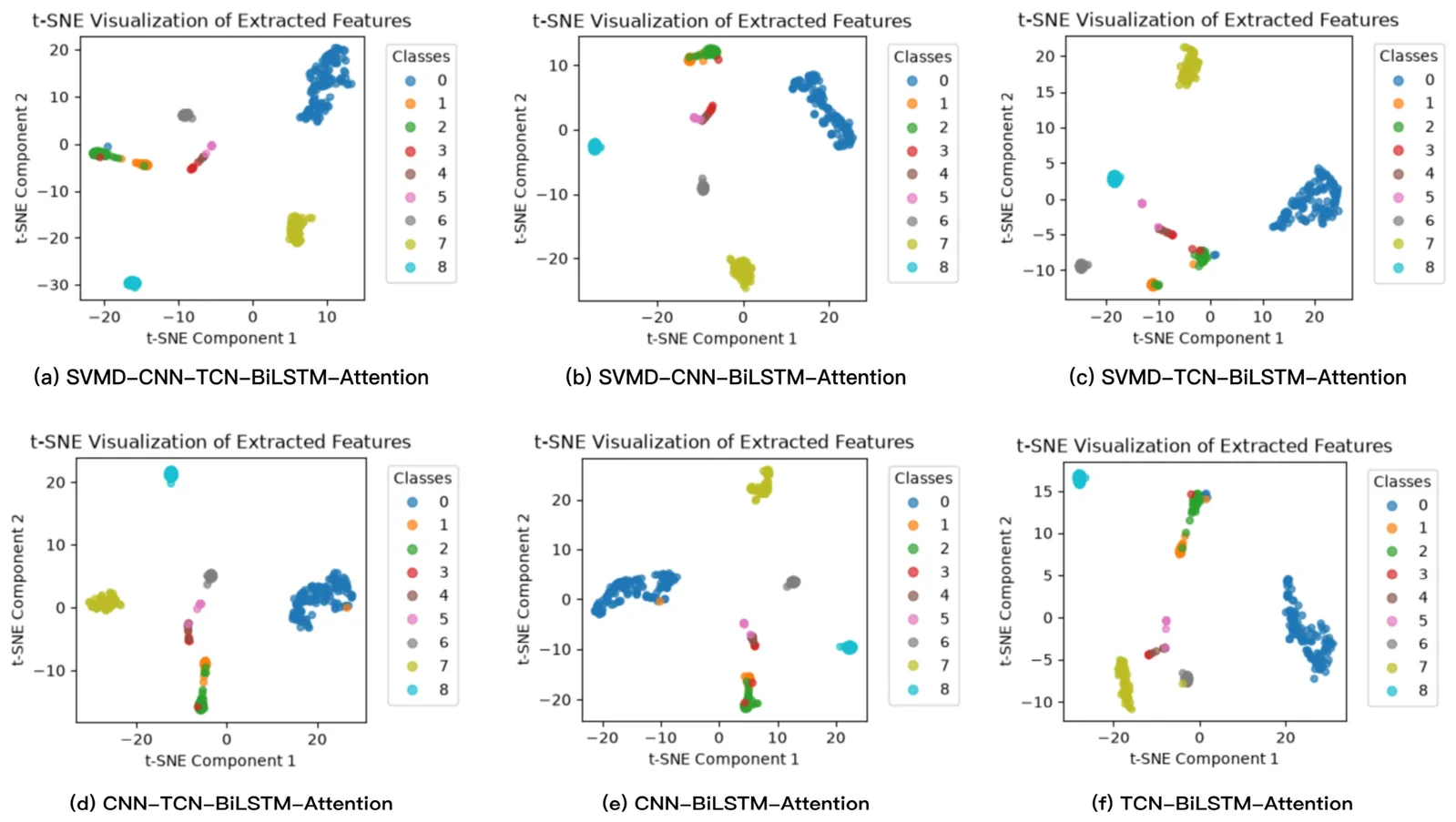
t-SNE visualization of learned feature representations from different fault diagnosis models.

t-SNE (t-distributed stochastic neighbor embedding) is a widely used dimensionality reduction technique for visualizing high-dimensional feature representations. It projects samples from a high-dimensional feature space into a two-dimensional space, allowing the distribution and separability of different classes to be intuitively observed. In this study, t-SNE visualization is employed to evaluate the discriminative capability of the features learned by different fault diagnosis models. The corresponding visualization results on the test set are presented in Figure 9.

As shown in Figure 9, all models achieve a certain degree of clustering for most fault categories, indicating that the extracted features contain effective fault-related information. Among them, the feature clusters corresponding to F0, F6, F7, and F8 exhibit high compactness and clear separation from other categories for all models. This result is consistent with the classification accuracy reported in Table 7, further confirming that these operating states possess highly distinguishable feature characteristics.

However, some fault categories still exhibit overlapping feature distributions. Specifically, the clusters of F1 and F2 show partial overlap, while F3, F4, and F5 also present relatively close distributions in the feature space. These observations indicate that these fault conditions share similar feature representations, which increases the difficulty of classification and is consistent with the misclassification patterns observed in the confusion matrices.

Compared with the other models, the proposed framework generates more compact intra-class clusters and clearer inter-class boundaries, with reduced overlap among different fault categories. This demonstrates that the proposed method can learn more discriminative feature representations by integrating signal denoising, spatiotemporal feature extraction, and attention-based feature enhancement. Consequently, the proposed framework achieves improved fault identification performance for multi-class PMSM fault diagnosis.

### 4.6. Comparison with Existing Representative Models in Terms of Performance and Computational Efficiency

To further validate the effectiveness of the proposed framework and compare it with representative existing methods, CNN-BiGRU-Attention, XGBoost + SMOTE, and RF + HGB + KNN were selected for comparison. CNN-BiGRU-Attention uses CNN for feature extraction, followed by BiGRU and multi-head attention to further extract useful feature information. XGBoost + SMOTE applies SMOTE during training to alleviate the class imbalance problem and then uses XGBoost for fault classification. RF + HGB + KNN adopts a late-fusion strategy to combine the prediction results of Random Forest, Histogram-based Gradient Boosting, and KNN. In addition to classification performance, the average inference time per sample and the total inference time on the test set were used to compare the inference efficiency of different models. The results are presented in Table 8.

As shown in Table 8, the proposed framework achieves the best fault classification performance, with an accuracy, precision, recall, and F1-Score of 0.985, outperforming all comparison models. CNN-BiGRU-Attention achieves the second-best overall performance, while XGBoost + SMOTE and RF + HGB + KNN show relatively lower classification performance. In terms of inference efficiency, the machine learning models require less inference time, with XGBoost + SMOTE showing the shortest average inference time. The proposed framework has the highest average inference time of 0.1435 ms/sample, indicating that the improved classification performance comes with a certain increase in computational cost.

Among the deep learning models, the proposed framework requires slightly more inference time than CNN-BiGRU-Attention, mainly due to the additional TCN branch and the associated feature extraction and fusion operations. However, the total inference time of the two models differs by only about 0.005 s, while the proposed framework achieves better fault classification performance. Overall, although the proposed framework has a relatively higher inference cost, its inference time remains short, with a total inference time of less than 0.05 s on the current test set. Therefore, under the experimental conditions considered in this study, the proposed framework provides a reasonable balance between fault classification performance and inference efficiency.

## 5. Conclusions

This study proposes a multi-sensor PMSM fault diagnosis framework for multi-class fault identification in inverter-driven PMSMs. The proposed framework integrates SVMD denoising, parallel CNN-TCN spatiotemporal feature extraction, and BiLSTM with multi-head attention to improve the representation of multi-sensor signals. Experiments on a public dataset containing eight sensor channels and nine operating states demonstrate that the proposed framework achieves an accuracy of 98.5% and outperforms the comparison models.

The experimental results show that SVMD denoising improves the quality of the input signals and contributes to better fault classification performance. The parallel CNN-TCN structure effectively combines spatial correlations and temporal dependencies, providing more comprehensive feature representations than using CNN or TCN alone. These results demonstrate the effectiveness of combining signal denoising, spatiotemporal feature extraction, and attention-based feature enhancement for multi-class PMSM fault diagnosis.

The main limitation of this study is that the evaluation is based on a public dataset collected under a fixed motor speed, DC supply voltage, and ambient temperature, rather than experiments on a physical PMSM prototype. In addition, F3 remains relatively difficult to identify due to its similarity to other fault categories. Future work will focus on prototype-based validation under different speeds, voltages, temperatures, and load conditions, as well as incorporating vibration signals to further exploit heterogeneous sensor information and improve cross-condition generalization.

## Figures and Tables

**Table 1:** Distribution of each fault category in the PMSM inverter fault dataset.

Class	Location	Description	Samples	Proportion (%)
F0	-	Normal operating condition	4295	39.4
F1	S3	High-side open-circuit fault	692	6.4
F2	S6	Low-side open-circuit fault	1122	10.3
F3	S2	Low-side short-circuit fault	407	3.7
F4	S3	High-side short-circuit fault	341	3.1
F5	S5	High-side short-circuit fault	412	3.8
F6	HB1	Overheating fault	854	7.9
F7	HB1 & HB2	Overheating fault	1735	15.9
F8	HB3	Overheating fault	1034	9.5

**Table 2:** Description of raw sensor measurements and selected derived features.

Feature	Description	Unit	Type
Ia	Phase A inline current	A	Raw measurement
Ib	Phase B inline current	A	Raw measurement
VDC	DC bus voltage	V	Raw measurement
IDC	DC bus current	A	Raw measurement
T1	Half bridge 1 temperature	°C	Raw measurement
T2	Half bridge 2 temperature	°C	Raw measurement
T3	Half bridge 3 temperature	°C	Raw measurement
VD	Driver voltage	V	Raw measurement
Ia_original	Phase A original currents	A	Derived feature
Ib_original	Phase B original currents	A	Derived feature
Power_DC	DC power (VDC × IDC_arduino)	W	Derived feature
Power_AC	AC power	W	Derived feature
Current_Imbalance	Phase current imbalance	-	Derived feature
Temp_Diff_Max	Maximum temperature difference	°C	Derived feature
VDC_RateOfChange	Rate of change of VDC	V/s	Derived feature
IDC_RateOfChange	Rate of change of IDC	A/s	Derived feature
VDC_MovingAvg	10-point moving average of VDC	V	Derived feature
IDC_MovingAvg	10-point moving average of IDC	A	Derived feature

**Table 3:** Pearson correlation coefficients of IMF components and corresponding signal reconstruction results after SVMD decomposition.

Feature	IMF_1	IMF_2	IMF_3	IMF_4	Reconstructed Signal
Ia	0.959	0.086	0.285	-	IMF_1 + IMF_3
Ib	0.962	0.080	0.223	-	IMF_1 + IMF_3
T1	0.999	0.032	0.086	-	IMF_1
T2	0.996	0.088	0.049	-	IMF_1
T3	0.994	0.096	0.223	-	IMF_1 + IMF_3
Ia_original	0.958	0.063	0.283	0.096	IMF_1 + IMF_3
Ib_original	0.962	0.063	0.202	0.094	IMF_1 + IMF_3
Temp_Diff_Max	0.997	0.039	0.043	-	IMF_1

**Table 4:** Sensitivity analysis of different Pearson correlation thresholds.

Pearson Threshold	Accuracy	Precision	Recall	F1-Score
0.2	0.959	0.962	0.959	0.959
0.1	0.985	0.985	0.985	0.985
0.08	0.969	0.971	0.969	0.969
0 (No Filtering)	0.972	0.973	0.972	0.972

**Table 5:** Detailed configurations of the proposed PMSM fault diagnosis model.

Block	Layer	Configuration
Input	Input Layer	Input size: 15 × 18
CNN Block	Conv1D + BN + ReLU + Dropout	16 filters, kernel size = 3, dropout = 0.2, MaxPool
	Conv1D + BN + ReLU + Dropout	32 filters, kernel size = 3, dropout = 0.2, MaxPool
TCN Block	Dilated Conv1D + Chomp + BN + ReLU + Dropout	16 filters, kernel size = 3, dilation = 1, dropout = 0.2
	Dilated Conv1D + Chomp + BN + ReLU + Dropout	32 filters, kernel size = 3, dilation = 2 dropout = 0.2
Fusion Block	Conv1D + BN + ReLU + Dropout	64 filters, kernel size = 1, dropout = 0.2
BiLSTM Block	BiLSTM layer 1	64 units × 2 directions, return sequences = True, dropout = 0.2
	BiLSTM layer 2	32 units × 2 directions, return sequences = True, dropout = 0.2
Attention Block	Multi-head Self-Attention	4 heads, key dimension = 16, LayerNorm, dropout = 0.2
	Feed-Forward Network	64 units, ReLU, LayerNorm, dropout = 0.2
Classifier Head	Global Average Pooling	-
	Dense + Dropout	64 units, dropout = 0.2
	Dense + Dropout	32 units, dropout = 0.2
Output	Dense	9 units, Softmax

**Table 6:** Overall performance comparison of different fault diagnosis models.

No.	Model	Accuracy	Precision	Recall	F1-Score
1	Proposed framework	0.985	0.985	0.985	0.985
2	SVMD-CNN-BiLSTM-Attention	0.975	0.978	0.975	0.975
3	SVMD-TCN-BiLSTM-Attention	0.966	0.968	0.966	0.966
4	CNN-TCN-BiLSTM-Attention	0.972	0.973	0.972	0.972
5	CNN-BiLSTM-Attention	0.963	0.965	0.963	0.962
6	TCN-BiLSTM-Attention	0.960	0.965	0.960	0.961

**Table 7:** Per-class classification accuracy of different fault diagnosis models.

No.	Model	F0	F1	F2	F3	F4	F5	F6	F7	F8
1	Proposed framework	0.984	0.952	0.909	0.750	0.900	0.917	1.000	1.000	1.000
2	SVMD-CNN-BiLSTM-Attention	1.000	0.857	0.969	0.833	1.000	0.833	1.000	1.000	1.000
3	SVMD-TCN-BiLSTM-Attention	0.953	0.952	0.939	0.833	1.000	0.917	1.000	1.000	1.000
4	CNN-TCN-BiLSTM-Attention	1.000	0.905	0.909	0.833	0.900	0.917	1.000	1.000	1.000
5	CNN-BiLSTM-Attention	1.000	0.857	0.939	0.583	0.900	0.917	1.000	1.000	1.000
6	TCN-BiLSTM-Attention	0.953	0.905	0.969	0.916	0.900	0.917	1.000	0.981	1.000

**Table 8:** Performance and computational efficiency comparison with existing representative models.

No.	Model	Accuracy	Precision	Recall	F1-Score	Average Inference Time (ms/Sample)	Total Inference Time (s)
1	Proposed framework	0.985	0.985	0.985	0.985	0.1435	0.0465
2	CNN-BiGRU-Attention	0.965	0.967	0.965	0.965	0.127	0.0411
3	XGBoost + SMOTE	0.956	0.959	0.956	0.957	0.0473	0.0153
4	RF + HGB + KNN	0.932	0.944	0.932	0.935	0.0735	0.0238

## Data Availability

The dataset used in this study is publicly available at https://doi.org/10.5281/zenodo.14482932.

## References

[1] Sergakis A., Salinas M., Gkiolekas N., Gyftakis K.N. (2025). A Review of Condition Monitoring of Permanent Magnet Synchronous Machines: Techniques, Challenges and Future Directions. Energies.

[2] Chen Y., Ren T., Li Y., Jiang G., Liu Q., Chen Y., Yang S.X. (2026). AI-empowered intelligence in industrial robotics: Technologies, challenges, and emerging trends. Intell. Robot..

[3] Sun S., Huang H., Li C. (2025). Advancements in humanoid robot dynamics and learning-based locomotion control methods. Intell. Robot..

[4] Minaz M.R. (2020). An effective method for detection of stator fault in PMSM with 1D-LBP. ISA Trans..

[5] Hasan Ebrahimi S., Choux M., Huynh V.K. (2022). Real-time detection of incipient inter-turn short circuit and sensor faults in permanent magnet synchronous motor drives based on generalized likelihood ratio test and structural analysis. Sensors.

[6] Jeong H., Lee H., Kim S., Kim S.W. (2022). Interturn short fault diagnosis using magnitude and phase of currents in permanent magnet synchronous machines. Sensors.

[7] Faiz J., Mazaheri-Tehrani E. (2016). Demagnetization modeling and fault diagnosing techniques in permanent magnet machines under stationary and nonstationary conditions: An overview. IEEE Trans. Ind. Appl..

[8] Attaianese C., D’Arpino M., Di Monaco M., Di Noia L.P. (2022). Model-based detection and estimation of DC offset of phase current sensors for field oriented PMSM drives. IEEE Trans. Ind. Electron..

[9] Chen Z., Liang D., Jia S., Yang S. (2024). Model-based data normalization for data-driven PMSM fault diagnosis. IEEE Trans. Power Electron..

[10] Abood S., Annamalai A., Chouikha M., Nejress T. (2025). Fault diagnostics of synchronous motor based on artificial intelligence. Machines.

[11] Jiang W., Wu J., Yang Y., Li X., Zhu H. (2025). Health evaluation techniques towards rotating machinery: A systematic literature review and implementation guideline. Reliab. Eng. Syst. Saf..

[12] de las Morenas J., Belmonte L.M., Morales R. (2025). Streamlined bearing fault detection using artificial intelligence in permanent magnet synchronous motors. Machines.

[13] Pietrzak P., Wolkiewicz M. (2022). Machine learning-based stator current data-driven PMSM stator winding fault diagnosis. Sensors.

[14] Orlowska-Kowalska T., Wolkiewicz M., Pietrzak P., Skowron M., Ewert P., Tarchala G., Krzysztofiak M., Kowalski C.T. (2022). Fault diagnosis and fault-tolerant control of PMSM drives—State of the art and future challenges. IEEE Access.

[15] Mesai Belgacem A., Hadef M., Ali E., Elsayed S.K., Paramasivam P., Ghoneim S.S. (2024). Fault diagnosis of inter-turn short circuits in PMSM based on deep regulated neural network. IET Electr. Power Appl..

[16] Pietrzak P., Wolkiewicz M. (2023). Fault diagnosis of PMSM stator winding based on continuous wavelet transform analysis of stator phase current signal and selected artificial intelligence techniques. Electronics.

[17] Cheng J., Ji F., Huang C., Wang T., Liu Y., Li Y. (2025). 1DCNN-residual bidirectional LSTM for permanent magnet synchronous motor temperature prediction based on operating condition clustering. IEEE Access.

[18] Wu Z., Huang N.E. (2009). Ensemble empirical mode decomposition: A noise-assisted data analysis method. Adv. Adapt. Data Anal..

[19] Torres M.E., Colominas M.A., Schlotthauer G., Flandrin P. (2011). A complete ensemble empirical mode decomposition with adaptive noise. Proceedings of the 2011 IEEE International Conference on Acoustics, Speech and Signal Processing (ICASSP).

[20] Chen Y., Zhu X., Wang P., Hao K., Sheng K. (2025). Adaptive variational empirical mode decomposition aware intelligent data-driven modeling for complex industrial processes. Intell. Robot..

[21] Li H., Shi T., Yan Y., Xia C. (2025). A method of prior feature extraction based on successive sparse estimation for inter-turn short circuit faults in PMSM. Adv. Eng. Inform..

[22] Canseven H.T., Sadık E.Ş., Cömert M., Ünsal A. (2026). Fault Detection and Diagnosis for Multi-Faults of PMSM-Drive Systems Using a Hybrid Machine Learning Method. IET Power Electron..

[23] Skowron M., Orlowska-Kowalska T., Kowalski C.T. (2021). Application of simplified convolutional neural networks for initial stator winding fault detection of the PMSM drive using different raw signal data. IET Electr. Power Appl..

[24] Mueller P.N., Woelfl L., Can S. (2023). Bridging the gap between AI and the industry—A study on bearing fault detection in PMSM-driven systems using CNN and inverter measurement. Eng. Appl. Artif. Intell..

[25] Tang M., Liang L., Zheng H., Chen J., Chen D. (2024). Anomaly detection of permanent magnet synchronous motor based on improved DWT-CNN multi-current fusion. Sensors.

[26] Yu Y., Yuan C., Zeng D., Carbone G., Hu Y., Yang J. (2024). Conceptual approach to permanent magnet synchronous motor turn-to-turn short circuit and uniform demagnetization fault diagnosis. Actuators.

[27] Yatak M.Ö. (2025). Inverter-Driven and Stator Winding Fault Detection in Permanent Magnet Synchronous Motors with Hybrid Deep Model. Electronics.

[28] Yılmaz Ü. (2026). Behavioral Fault Diagnosis in Inverter-Driven PMSM Systems Using a Hybrid CNN-BiLSTM-Attention Deep Learning Framework with SHAP-Based Interpretability. Machines.

[29] Wang J., Ma J., Meng D., Zhao X., Zhang K. (2023). Fault diagnosis of PMSMs based on image features of multi-sensor fusion. Sensors.

[30] Zhu M., Hu W., Kar N.C. (2018). Multi-sensor fusion-based permanent magnet demagnetization detection in permanent magnet synchronous machines. IEEE Trans. Magn..

[31] Song J., Li F., Zhao J., Wang L., Liu B., Wang X., Lu S. (2025). PMSLM Demagnetization Fault Detection Based on Multi-Sensor Signal Fusion and Enhanced Graph Neural Network. IEEE Trans. Transp. Electrif..

[32] Wang X., Yang B., Li A., Lu S., Zhang Y., Song J. (2026). PMSM High-Resistance Connection Fault Diagnosis via Deep Feature Fusion of Multi-Sensor Signals. IEEE Sens. J..

[33] Nazari M., Sakhaei S.M. (2020). Successive variational mode decomposition. Signal Process..

[34] Li Y., Ding W., Mao Y. (2026). An Adaptive Enhancement Method for Weak Fault Diagnosis of Locomotive Gearbox Bearings Under Wheel-Raisl Excitation. Machines.

[35] Zhang L., Xu Y., Xue H., Zhu C., Xu Z. (2026). Fault Diagnosis Method for Electric Vehicle In-Wheel Motor Bearings Based on Improved SVMD and ResNet-KAN. Sensors.

[36] Ammar K., Fraihat S., Al-Naymat G., Sanjalawe Y. (2025). ECG-CBA: An End-to-End Deep Learning Model for ECG Anomaly Detection Using CNN, Bi-LSTM, and Attention Mechanism. Algorithms.

[37] Aversano L., Mancino I., Marengo A., Verdone C. (2026). Comparative Analysis of Machine Learning and Deep Learning Models for Atrial Fibrillation Detection from Long-Term ECG. Appl. Sci..

[38] Bai S., Kolter J.Z., Koltun V. (2018). An empirical evaluation of generic convolutional and recurrent networks for sequence modeling. arXiv.

[39] Wu H., Yang Y., Chen W., Wang Y. (2025). A New Asymmetric Track Filtering Algorithm Based on TCN-ResGRU-MHA. Symmetry.

[40] Xing J., Fang X., Bai J., Cui L., Zhang F., Xu Y. (2026). Study on Multimodal Sensor Fusion for Heart Rate Estimation Using BCG and PPG Signals. Sensors.

[41] Torres N.N.S., Maciel J.N., Lima T.L.d.V., Gazziro M., Filho A.C.L., Carmo J.P.P.d., Ando Junior O.H. (2025). Fault Diagnosis in Internal Combustion Engines Using Artificial Intelligence Predictive Models. Appl. Syst. Innov..

[42] Choi S.-G., Seo S.-H., Yu J.-H., Choi Y.-J., Tong K.-C., Choi M.-H., Jung Y.-G., Baek M.-S., Song H.-K. (2025). A Transformer-Based Approach for Joint Interference Cancellation and Signal Detection in FTN-RIS MIMO Systems. Mathematics.

[43] Bacha A., El Idrissi R., Idrissi K.J., Lmai F. (2025). Comprehensive dataset for fault detection and diagnosis in inverter-driven permanent magnet synchronous motor systems. Data Brief.

